# A prognostic nomogram for the cancer-specific survival of patients with upper-tract urothelial carcinoma based on the Surveillance, Epidemiology, and End Results Database

**DOI:** 10.1186/s12885-020-07019-5

**Published:** 2020-06-08

**Authors:** Chengzhuo Li, Jin Yang, Fengshuo Xu, Didi Han, Shuai Zheng, Rahel Elishilia Kaaya, Shengpeng Wang, Jun Lyu

**Affiliations:** 1 Department of Clinical Research, The First Affiliated Hospital of Jinan University, Guangzhou, Guangdong China; 2 School of Public Health, Xi’an Jiaotong University Health Science Center, Xi’an, Shaanxi China; 3 School of Public Health, Shaanxi University of Chinese Medicine, Xi’an, Shaanxi China; 4 Cardiovascular Research Center, School of Basic Medical Sciences, Xi’an Jiaotong University Health Science Center, Xi’an, 710061 People’s Republic of China; 5 Key Laboratory of Environment and Genes Related to Diseases of Ministry of Education, Xi’an Jiaotong University Health Science Center, Xi’an, Shaanxi China

**Keywords:** Upper-tract urothelial carcinoma, Nomogram, SEER, Cancer-specific survival

## Abstract

**Background:**

The aim of this study was to establish a comprehensive nomogram for the cancer-specific survival (CSS) of patients with upper-tract urothelial carcinoma (UTUC) and compare it with the traditional American Joint Committee on Cancer (AJCC) staging system in order to determine its reliability.

**Methods:**

This study analyzed 9505 patients with UTUC in the Surveillance, Epidemiology, and End Results (SEER) database. R software was used to randomly divided the patients in a 7-to-3 ratio to form a training cohort (*n* = 6653) and a validation cohort (*n* = 2852). Multivariable Cox regression was used to identify predictive variables. The new survival model was compared with the AJCC prognosis model using the concordance index (C-index), the area under the time-dependent receiver operating characteristics curve (AUC), the net reclassification improvement (NRI), the integrated discrimination improvement (IDI), calibration plotting, and decision-curve analysis (DCA).

**Results:**

We have established a nomogram for determining the 3-, 5-, and 8-year CSS probabilities of UTUC patients. The nomogram indicates that the AJCC stage has the greatest influence on CSS in UTUC, followed by the age at diagnosis, surgery status, tumor size, radiotherapy status, histological grade, marital status, chemotherapy status, race, and finally sex. The C-index was higher for the nomogram than the AJCC staging system in both the training cohort (0.785 versus 0.747) and the validation cohort (0.779 versus 0.739). Calibration plotting demonstrated that the model has good calibration ability. The AUC, NRI, IDI, and DCA of the nomogram showed that it performs better than the AJCC staging system alone.

**Conclusions:**

This study is the first to establish a comprehensive UTUC nomogram based on the SEER database and evaluate it using a series of indicators. Our novel nomogram can help clinical staff to predict the 3-, 5-, and 8-year CSS probabilities of UTUC patients more accurately than using the AJCC staging system.

## Background

Urothelial carcinoma is a type of urinary tumor that can occur in the upper urinary tract (renal pelvis and ureter) or the lower urinary tract (bladder and urethra). Although urothelial carcinoma is the fourth most common type of tumor [1], upper-tract urothelial carcinoma (UTUC) is a rare malignancy of the urinary system that accounts for about 10% of all renal tumors and 5% of all urothelial tumors [2]. UTUC includes carcinoma of the renal pelvis and ureter, and ureteral tumors are less common than renal pelvis tumors [3]. Most of the few studies that have investigated UTUC have combined UTUC with kidney cancer. However, since the incidence and mortality rates of UTUC have increased in recent years [3–5], the present study focused on analyzing UTUC alone.

Age at diagnosis and being male are known risk factors for UTUC [3]. Surgery is the preferred approach for treating UTUC, and nephroureterectomy with bladder cuff excision has been the mainstay treatment [6]. The roles of chemotherapy and radiotherapy in advanced disease have not been clearly demonstrated, but some studies have found chemotherapy to be beneficial [7]. UTUC has a more-aggressive clinical course and a worse prognosis than bladder cancer [8], and the currently available prognostic models of UTUC are inadequate.

The traditional American Joint Committee on Cancer (AJCC) staging system provides clinically significant prognoses of UTUC and is currently the main reference standard for the prognosis of clinical treatment [9]. However, the AJCC staging system does not incorporate the entire pathological nature of the tumor, excluding potentially important factors when predicting the prognosis such as demographic characteristics, tumor size, tumor location, and the treatment applied [10–12]. A nomogram is based on a prognostic model and it can clearly and concisely show how various prognostic factors influence certain outcome variables. A nomogram can be used to calculate the survival probability of individual patients, making it of great value in clinical practice [13].

The Surveillance, Epidemiology, and End Results (SEER) database has not previously been used to construct a prognostic nomogram for UTUC. Therefore, the purpose of this study was to establish a comprehensive nomogram that includes both demographic factors and clinicopathological features. The new prediction model was compared with the traditional AJCC staging system in order to determine its reliability. The developed nomogram has considerable clinical value in helping clinical staff to predict the 3-, 5-, and 8-year cancer-specific survival (CSS) probability of UTUC patients more comprehensively and on an individual basis.

## Methods

### Source of data

We analyzed data obtained from the SEER database. Part of that database is open to the public, and we also searched for additional chemotherapy data using the SEER*Stat software [14, 15]. We extracted UTUC patients from the SEER database in the following ways: [16] The primary sites of UTUC were selected using the codes “C65.9-Renal pelvis” and “C66.9-Ureter.” All of the ICD-O-3 histology and behavior codes related to UTUC were included. Age at diagnosis, race, sex, and marital status were selected as demographic characteristics. The following pathological features were also included: primary site, histological grade, AJCC stage, tumor size, surgery status, radiotherapy status, and chemotherapy status. It is worth noting that the tumor histological grade is divided into four levels in the SEER database. The four-grade system describes the tumor as Grade I: well-differentiated; Grade II: moderately differentiated; Grade III: poorly differentiated; Grade IV: undifferentiated or anaplastic. We chose the AJCC stage based on the sixth edition of the Derived AJCC Stage Group. The tumor size was divided into three categories based on the diameter: < 2, 2–4, and > 4 cm [1, 17]. We classified the surgery status based on the records in the SEER database. “Yes” means surgery performed. “No” means three situations: patient died prior to recommended surgery, not recommended, and recommended but patient refused. The outcome in the study was death due to UTUC.

### Criteria for data selection

**Fig. 1:**
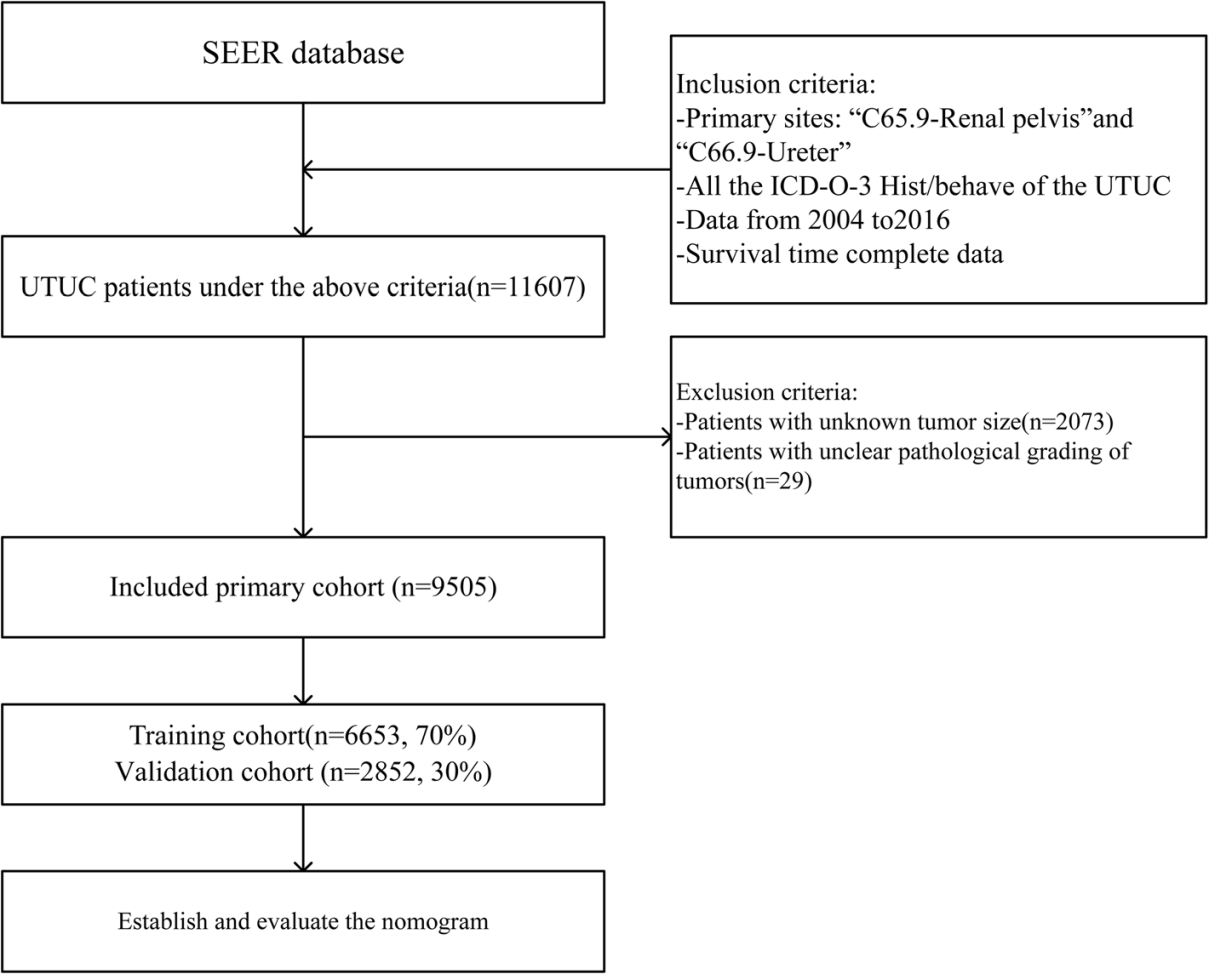
Research flowchart

This retrospective study initially identified 11,607 UTUC patients enrolled in the SEER database between 2004 and 2016 by applying the above criteria. However, 2073 patients were not included in the analysis due to the tumor size being unknown, with a further 29 patients rejected due to unclear histological tumor grading. Thus, we finally selected 9505 UTUC patients, and classified 70% (*n* = 6653) of them into the training cohort for constructing the prognostic nomogram and 30% (*n* = 2852) of them into the validation cohort for evaluating the constructed nomogram. The data screening process is shown in Fig. 1.

### Statistical analysis

We performed a descriptive analysis of all of the above-mentioned factors. The age at diagnosis was expressed as median and interquartile-range values, while other categorical variables were represented as percentages. Cox regression was used to screen for correlation factors for which *p* = 0.1. We then established a nomogram that predicted the 3-, 5-, and 8-year CSS probabilities of UTUC.

After establishing the nomogram, we used a series of indicators to evaluate it. We first used the concordance index (C-index) and the area under the time-dependent receiver operating characteristics (ROC) curve (AUC) to evaluate the differentiation ability of the new model, and then supplemented this by adopting two relatively new indicators (net reclassification improvement [NRI] and integrated discrimination improvement [IDI]) to increase the accuracy and comprehensiveness of the comparisons [18, 19]. The consistency of survival probabilities predicted using the nomogram with the actual situation was evaluated by drawing calibration plots [20]. Finally, we used decision-curve analysis (DCA) to evaluate the clinical validity of the model [21].

All of the statistical analyses were performed using IBM SPSS Statistics software (version 23.0, SPSS, Chicago, IL, USA) and R software (version 3.4.1; http://www.Rproject.org). R software was used to randomly divide the 9505 patients in a 7-to-3 ratio to the 2 study cohorts, and the log-rank test was also used to check that there were no significant differences between the cohorts. A bilateral probability value of *p* < 0.05 was considered indicative of statistical significance.

It is not necessary to obtain informed patient consents for data obtained from the SEER database since it does not include information that can be used to identify individual patients.

## Results

### Characteristics of the included patients

The median age at diagnosis was 73 years (interquartile range, 65–80 years) in the training and validation cohorts. Most of the patients in the training and validation cohorts were male (59.2 and 60.0%, respectively), white (87.7 and 87.3%), and married (87.5 and 87.9%). Among the tumor-related features, the primary site was predominantly in the renal pelvis (65.7 and 66.6% in the training and validation cohorts, respectively), with the remainder in the ureter. Most of the tumors were at histological grade IV and larger than 4 cm in both cohorts. The distribution of the different AJCC stages was close to uniform. Most of the patients had received surgery, with only a few receiving radiotherapy or chemotherapy in both cohorts. Table 1 summarizes the demographic and tumor characteristics of the two cohorts.

**Table 1:** Socio-demographic and clinical characteristics of patients in the study

Variable	Training Cohort	Validation Cohort	*P*-value
Number of Patients n (%)	6653 (70)	2852 (30)	
Age of diagnosis	73 (65–80)	73 (65–80)	0.891
Sex n (%)			0.457
Male	3937 (59.2)	1711 (60.0)	
Female	2716 (40.8)	1141 (40.0)	
Race n (%)			0.442
White	5832 (87.7)	2489 (87.3)	
Black	279 (4.2)	136 (4.8)	
Other	542 (8.1)	227 (8.0)	
Marital status n (%)			0.844
Married	5820 (87.5)	2507 (87.9)	
Unmarried	577 (8.7)	238 (8.3)	
Other	256 (3.8)	107 (3.8)	
Site n (%)			0.412
Renal pelvis	4372 (65.7)	1899 (66.6)	
Ureter	2281 (34.3)	953 (33.4)	
Grade n (%)			0.538
I	287 (4.3)	126 (4.4)	
II	989 (14.9)	406 (14.2)	
III	1990 (29.9)	850 (29.8)	
IV	3387 (50.9)	1470 (51.5)	
Size n (%)			0.003
< 2	1042 (15.7)	413 (14.5)	
[2,4)	2608 (39.2)	1053 (36.9)	
≥ 4	3003 (45.1)	1386 (48.6)	
AJCC stage n (%)			0.901
I	1900 (28.6)	836 (29.3)	
II	1104 (16.6)	462 (16.2)	
III	2125 (31.9)	882 (30.9)	
IV	1524 (22.9)	672 (23.6)	
Surgery n (%)			0.075
Yes	6359 (95.6)	2702 (94.7)	
NO/Unknown	294 (4.4)	150 (5.3)	
Radiotherapy n (%)			0.942
Yes	357 (5.4)	152 (5.3)	
NO/Unknown	6296 (94.6)	2700 (94.7)	
Chemotherapy n (%)			0.217
Yes	1344 (20.2)	608 (21.3)	
NO/Unknown	5309 (79.8)	2244 (78.7)	

### Variable screening and nomogram establishment

The age at diagnosis, sex, race, marital status, primary site, histological grade, tumor size, AJCC stages, surgery status, radiotherapy status, and chemotherapy status were entered into the multivariable Cox regression analysis. The results showed that all of the factors except the primary site were suitable for including in the model. The multivariable analysis revealed that the following factors were statistically significant: age at diagnosis (hazard ratio [HR] = 1.016, *p* < 0.001), female (HR = 1.144, *p* < 0.01 versus male), black (HR = 1.223, *p* = 0.088 versus white), other race (HR = 1.186, *p* < 0.05 versus white), unmarried (HR = 1.236, *p* < 0.05 versus married), histological grade III (HR = 1.661, *p* < 0.01 versus grade I), histological grade IV (HR = 1.791, *p* < 0.001 versus grade I), size = 2–4 cm (HR = 1.314, *p* < 0.01 versus size < 2 cm), size > 4 cm (HR = 1.831, *p* < 0.001 versus size < 2 cm), AJCC stage II (HR = 1.609, *p* < 0.001 versus stage I), AJCC stage III (HR = 2.881, *p* < 0.001 versus stage I), AJCC stage IV (HR = 8.674, *p* < 0.001 versus stage I), no/unknown surgery status (HR = 2.936, *p* < 0.001 versus surgery), no/unknown radiotherapy status (HR = 0.715, *p* < 0.001 versus radiotherapy), and no/unknown chemotherapy status (HR = 1.254, *p* < 0.001 versus chemotherapy). Table 2 lists the results of the multivariable Cox regression analysis.

**Table 2:** Selected variables by multivariable Cox regression analysis

Variable	Multivariable analysis		
	HR^a^	95%CI^b^	*P*-value
Age of diagnosis	1.016	1.011–1.021	0.000***
Sex			
Male	Reference		
Female	1.144	1.039–1.260	0.006**
Race			
White	Reference		
Black	1.223	0.970–1.542	0.088
Other	1.186	1.014–1.388	0.033*
Marital status			
Married	Reference		
Unmarried	1.236	1.048–1.458	0.012*
Other	0.907	0.691–1.190	0.480
Site			
Renal pelvis	Reference		
Ureter	1.073	0.963–1.195	0.200
Grade			
I	Reference		
II	1.029	0.709–1.494	0.879
III	1.661	1.175–2.346	0.004**
IV	1.791	1.271–2.522	0.000***
Size			
< 2	Reference		
2–4	1.314	1.096–1.577	0.003**
≥ 4	1.831	1.533–2.187	0.000***
AJCC stage			
I	Reference		
II	1.609	1.311–1.975	0.000***
III	2.881	2.433–3.411	0.000***
IV	8.674	7.278–10.338	0.000***
Surgery			
Yes	Reference		
NO/Unknown	2.936	2.481–3.475	0.000***
Radiotherapy			
Yes	Reference		
NO/Unknown	0.715	0.608–0.841	0.000***
Chemotherapy			
Yes	Reference		
NO/Unknown	1.254	1.112–1.413	0.000***

Notes: ∗*P* < 0.05; ∗∗*P* < 0.01; ∗∗∗*P* < 0.001

Abbreviations: ^a^*HR* hazard ratio, ^b^*CI* confidence interval

### Nomogram comparison and evaluation

**Fig. 2:**
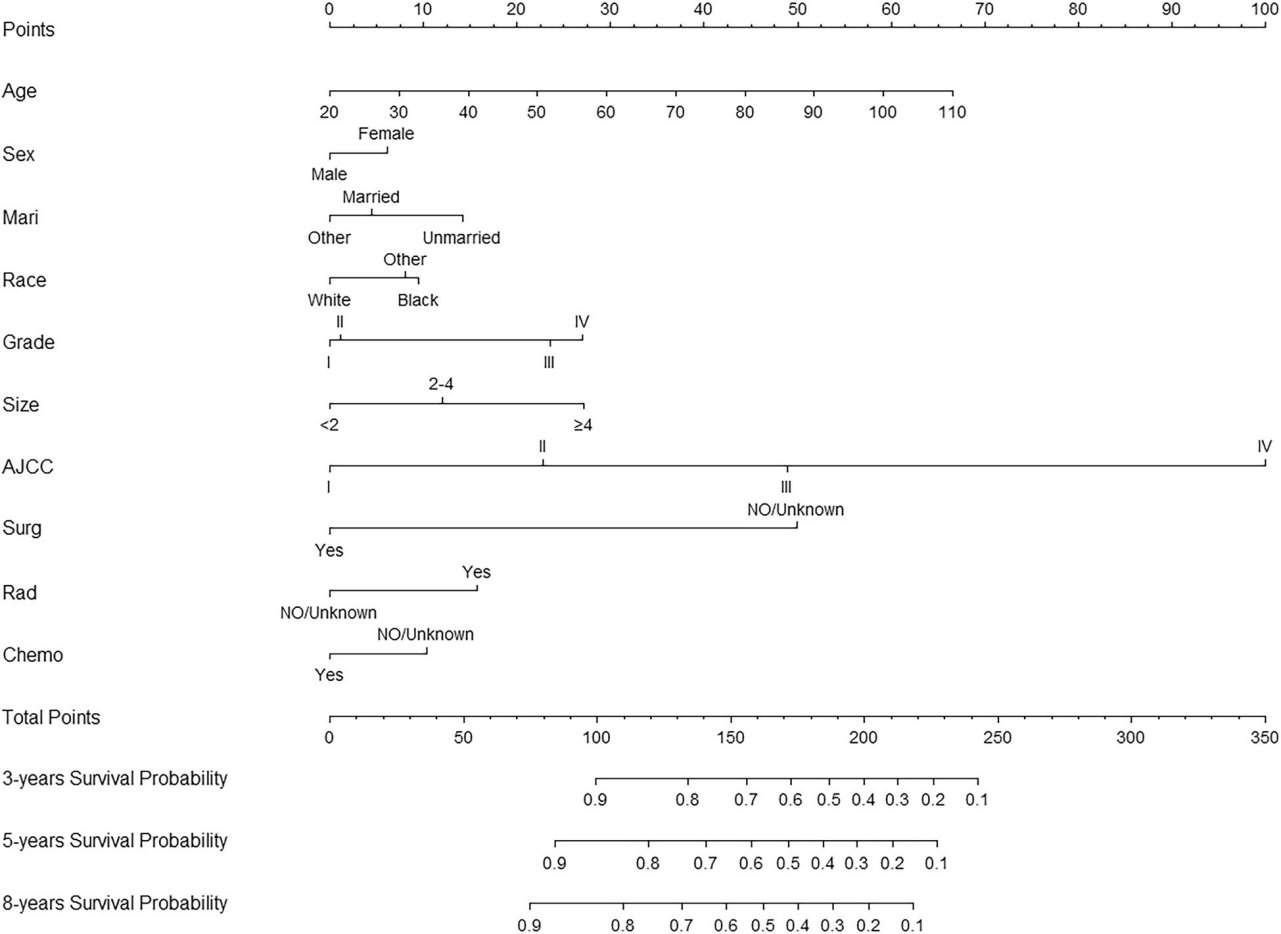
Nomogram predicting 3-, 5-, and 8-years CSS probability. Mari-marital status; Surg –surgery status; Rad – radiotherapy status; Chemo –chemotherapy status

Figure 2 shows the nomogram for predicting the 3-, 5-, and 8-year CSS probabilities for UTUC patients that we established based on the findings of the multivariable Cox regression analysis. It can be seen from the nomogram that the AJCC stage has the greatest influence on the CSS probability for UTUC, followed by age at diagnosis, surgery status, tumor size, radiotherapy status, histological grade, marital status, chemotherapy status, race, and finally sex.

**Fig. 3:**
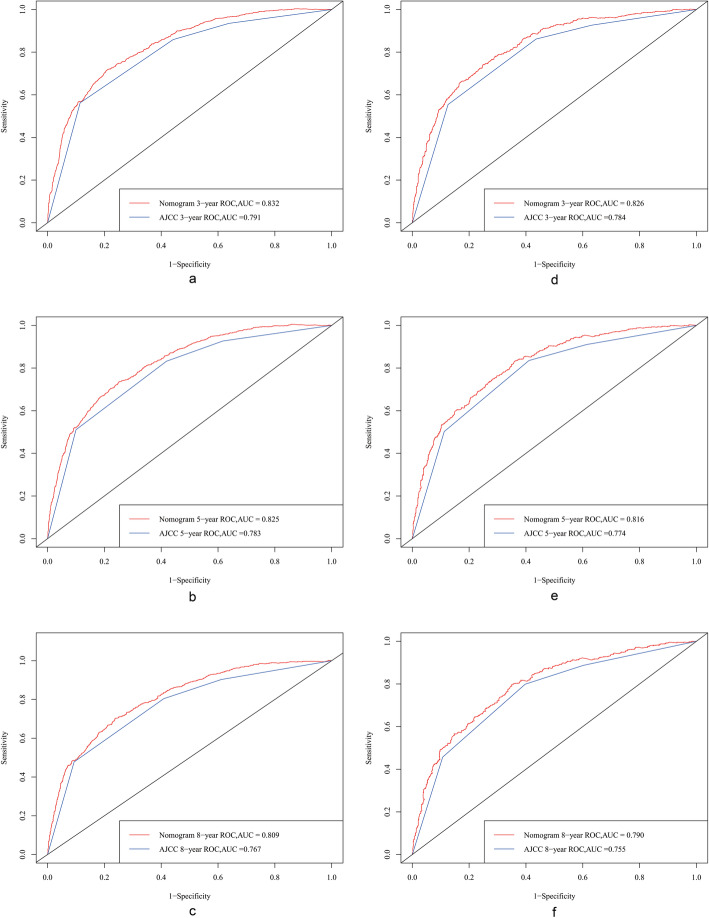
ROC curves. The area under the ROC curve (AUC) was used to evaluate the performance of the new nomogram. **a**, **b**, and **c** represent the result of the training cohort; **d**, **e**, and **f** represent the result of the validation cohort

After establishing the nomogram we used a series of indicators to evaluate the performance of the new prediction model underpinning this nomogram. We first used the C-index to evaluate the effect of the nomogram, and found that this was higher for the nomogram than for the AJCC staging system in both the training cohort (0.785 versus 0.747) and the validation cohort (0.779 versus 0.739). We further compared ROC curves, which revealed that in the training cohort the 3-, 5-, and 8-year AUC values were 0.832, 0.825, and 0.809, respectively, for the nomogram, which were all higher than those for the AJCC staging system (0.791, 0.783, and 0.767); the corresponding values for the validation cohort were 0.826, 0.816, 0.790, 0.784, 0.774, and 0.755, respectively (Fig. 3).

The NRI values for the 3-, 5-, and 8-year CSS probabilities were 0.219 (95% confidence interval [CI] = 0.171–0.297), 0.247 (95% CI = 0.180–0.332), and 0.259 (95% CI = 0.195–0.358), respectively, in the training cohort, and 0.259 (95% CI = 0.125–0.359), 0.272 (95% CI = 0.152–0.389), and 0.265 (95% CI = 0.148–0.393) in the validation cohort. In addition, the IDI values for the 3-, 5-, and 8-year CSS probabilities were 0.029, 0.028, and 0.026, respectively (*p* < 0.001), in the training cohort, 0.031, 0.026, and 0.025, respectively (*p* < 0.001), in the validation cohort. All of the NRI and IDI values being greater than zero indicate that the new model had better discrimination ability than the AJCC staging system.

**Fig. 4:**
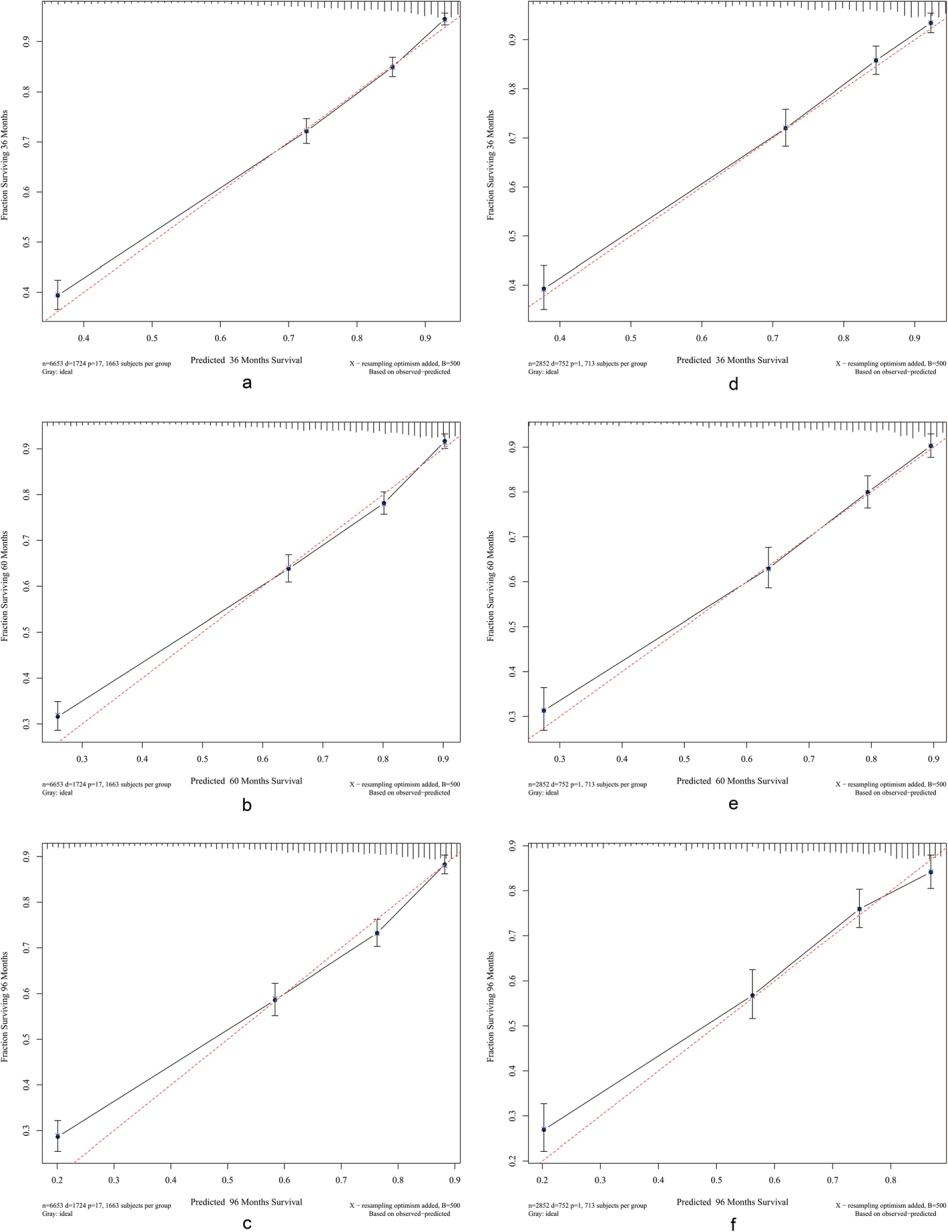
Calibration curves. Calibration curves for 3-, 5-, and 8-years cancer specific survival probability depict the calibration of each model in terms of the agreement between the predicted probabilities and observed outcomes of the training cohort (**a**, **b**, **c**) and validation cohort (**d**, **e**, **f**)

The calibration plots showed that the standard curves of the 3-, 5-, and 8-year CSS probabilities of the model was very close to the standard 45-degree diagonal lines and that the calibration points were evenly distributed, which demonstrated that the new model had good calibration ability (Fig. 4).

**Fig. 5:**
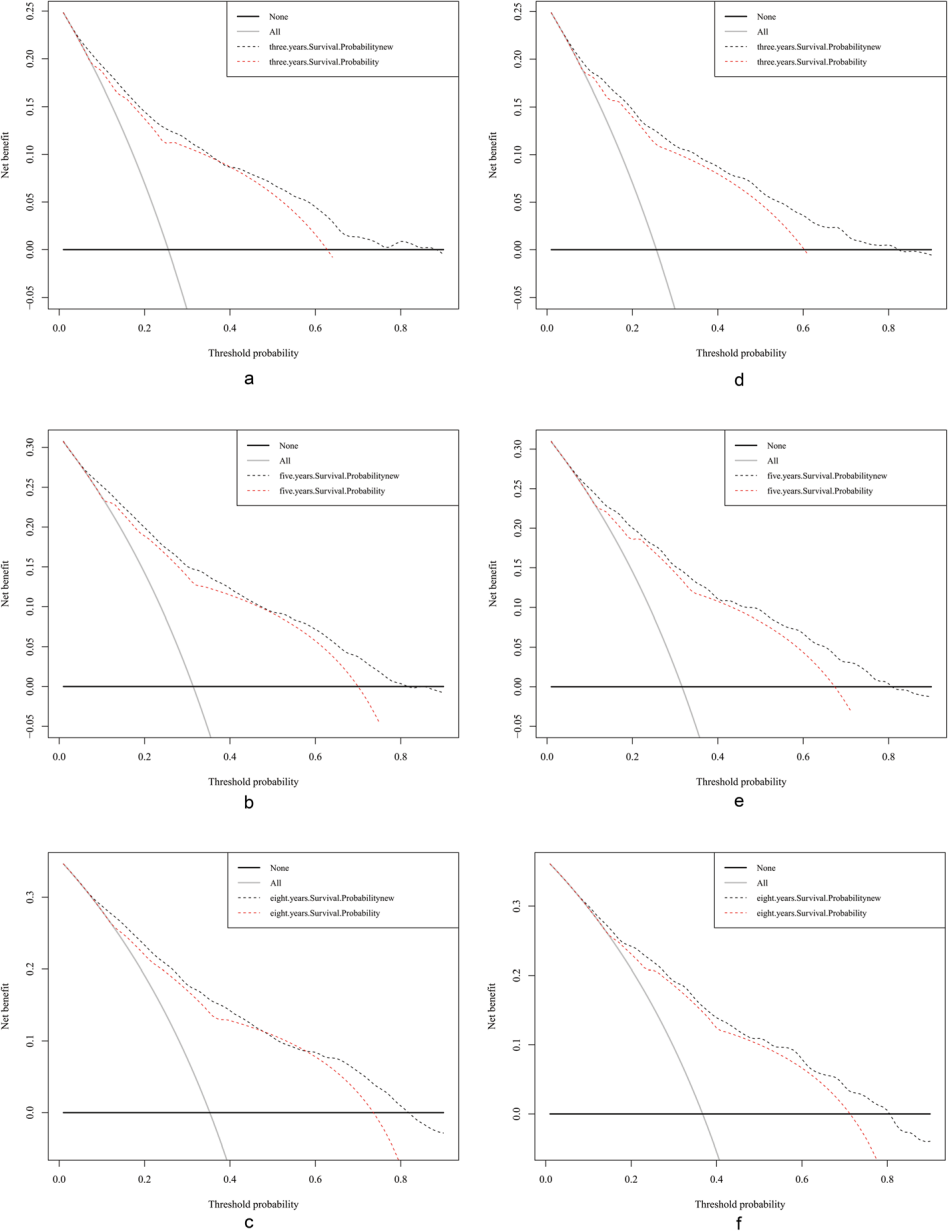
Decision curve analysis curves. Decision curve analysis of the training cohort (**a**, **b**, **c**) and validation cohort (**d**, **e**, **f**) for 3-, 5-, and 8-years cancer specific survival probability

The abscissa in DCA curves is the threshold probability and the ordinate is the net benefit after the benefit is subtracted from the disadvantage [22]. Compared with the AJCC staging system, the 3-, 5-, and 8-year DCA curves were found to be enhanced for both the training and validation cohorts (Fig. 5).

## Discussion

Previous studies of UTUC have been inadequate, with many clinical studies combining UTUC with renal or bladder cancer, which is not consistent with UTUC have its own unique pathological features [23]. The recent increases in the incidence and mortality rates of UTUC mean that the importance of determining the clinical prognosis of UTUC is also increasing [24]. The prognosis of UTUC is poor, and there is a lack of comprehensive and simple support research for this disease.

The special clinical characteristics of UTUC make it necessary to develop a UTUC-specific nomogram for providing more-accurate prediction models for use by clinical staff. In this study we successfully constructed a prognostic nomogram for UTUC patients using case data obtained from the SEER database. Nomograms are widely used in oncology and medicine to predict prognoses and meet the needs of clinical staff to provide patients with individualized treatments, and they are easier to understand than the traditional AJCC staging system [25]. The multivariable Cox regression analysis performed in the present study revealed that age at diagnosis, sex, race, marital status, histological grade, tumor size, AJCC stage, surgery status, radiotherapy status, and chemotherapy status are associated with the prognosis of UTUC.

One of the prognostic factors included in the new model, age, has long been considered a risk factor for UTUC [26]. In contrast, whether sex is a risk factor for UTUC had not been determined previously [27], but the present study found that being female is a risk factor for survival (HR = 1.142, *p* < 0.01). Moreover, our study found for the first time that being unmarried is a risk factor of CSS affecting the prognosis of UTUC. A study showed that unmarried patients are at significantly higher risk of presentation with metastatic cancer, undertreatment, and death resulting from their cancer [28]. The relationship between marriage and cancer prognosis may be due to the following reasons. First of all, married patients may be higher than unmarried in terms of economic level and education level. Married persons also have better adherence to treatment, which may lead to differences in the prognosis of different marital status [29]. Second, a study showed that married patients were less likely to present with metastatic disease than those who were unmarried [28]. Finally, a review confirmed that marriage positively influences the likelihood of early diagnosis for all types of cancer. Correspondingly, if an unmarried person is diagnosed with cancer, the risk of developing advanced disease is greater, and the life expectancy is usually shorter [30]. In short, the prognosis of unmarried patients in this study is poor, and more reminders should be given to unmarried patients in this regard.

Histological grade, AJCC stage, surgery status, radiotherapy status, and chemotherapy status were also found to affect the survival probability. However, it is worth noting that the survival probability decreased in UTUC patients who received radiotherapy, which is consistent with the findings of Leow et al. [7] However, it should be noted that the gold standard treatment for UTUC is still surgery. Radiation therapy is usually performed in patients who have progressed to the point where surgery cannot be performed [31]. The experimental research on radiotherapy alone is very limited, which is worthy of further research in UTUC. On the other hand, this is a retrospective study and there are selection biases that are difficult to adjust. Therefore, the exact relationship between radiotherapy and UTUC prognosis needs further prospective experiments to confirm. In addition, like some previous studies [32, 33], tumor size was included in our model as a risk factor. However, the tumor site was not included in the model, meaning that this does not affect the prognosis of UTUC. Figure 2 clearly shows the relevant factors and their effects on the 3-, 5-, and 8-year CSS probabilities in UTUC patients. The total score can be obtained by adding the individual scores for each of the above factors, and clinical staff can use this score to predict the CSS probability of individual patients and thereby make decisions that are more likely to improve their prognosis.

After constructing the nomogram and analyzing related prognostic factors, it was compared with traditional the AJCC model using a training cohort and an internal validation cohort in order to evaluate the model underlying the nomogram. We used the C-index and AUC to evaluate the discrimination performance, and found that both of these parameters were higher for the nomogram than for the AJCC staging system in both the training and validation cohorts (Fig. 3). When adding a new parameter to a model and then performing a comparison to see whether the predictive power of the model has improved, the increase in the AUC is sometimes not obvious. Instead, the NRI is often used to compare the prediction powers of two models, while the IDI can be used to reflect the overall model improvement [34, 35]. The NRI of the prediction model showed that after adding the new index, the proportion of correct classifications for the 3-, 5-, and 8-year survival probabilities increased by 21.9, 24.7, and 25.9%, respectively, in the training cohort, and by 25.9, 27.2, and 26.5% in the validation cohort (*p* < 0.001). The IDI revealed that the new model improved the predictive abilities for the 3-, 5-, and 8-year survival probabilities compared with the old model by 2.9, 2.8, and 2.6%, respectively, in the training cohort, and by 3.1, 2.6, and 2.5% in the validation cohort (*p* < 0.001).

We used calibration curves to evaluate the calibration performance of the model. The 45-degree line in Fig. 4 is the standard line [36]. The broken lines in the figure are very close to the standard line and the predicted points are evenly distributed, which indicates that the nomogram exhibited good discrimination and calibration abilities both in the training and validation cohorts.

DCA is a method to evaluate prediction models by calculating the clinical net benefit. Figure 5 shows that the DCA curves of the nomogram for the 3-, 5-, and 8-year survival probabilities were almost all above those for the traditional AJCC model, which means that the new model has better clinical effectiveness.

This study was subject to several limitations. The first limitation is that the study had a retrospective design and obtained data from the SEER database, which inevitably resulted in the presence of selection bias and information bias; for example, it is improper to integrate “no” and “unknown” into one category in the SEER database. The second limitation was that some potentially important factors were not included in the study, making it insufficiently comprehensive, such as certain biological indicators and behavioral habits. Finally, external validation of the nomogram was not performed, and the use of only internal validation may lead to overfitting of the new model. In the future we plan to incorporate more predictors and validate the effect of the model with external cohorts in order to obtain more-accurate results.

## Conclusion

This study is the first to establish a comprehensive UTUC nomogram based on the SEER database and evaluate it using a series of indicators. A particularly interesting finding was of the marital status being a prognostic factor for UTUC. The tumor size also significantly affected the prognosis, will the primary site of UTUC did not. Our novel nomogram can be used a tool to help clinical staff to predict the 3-, 5-, and 8-year CSS probabilities of UTUC patients more accurately than using the AJCC staging system.

## Data Availability

SEER collects cancer incidence data from population-based cancer registries covering approximately 34.6% of the U.S. population. SEER releases a standard set of research data every spring based on the previous November’s submission of data from the registries. The data we used is based on the November 2018 submission. We accessed these through the SEER*Stat software with additional approvals. The data that support the findings of this study are available from the SEER*Stat software but restrictions apply to the availability of these data, which were used under license for the current study, and so are not publicly available. Data are however available upon reasonable request and with the SEER Research Data Agreement.

## Abbreviations

UTUC: Upper-tract urothelial carcinoma
AJCC: American Joint Committee on Cancer
SEER: Surveillance, Epidemiology, and End Results
C-index: Concordance index
AUC: Area under the time-dependent receiver operating characteristic curve
NRI: Net reclassification improvement
IDI: Integrated discrimination improvement
DCA: Decision curve analysis
